# A dynamic clinical pathway for the treatment of patients with early breast cancer is a tool for better cancer care: implementation and prospective analysis between 2002–2010

**DOI:** 10.1186/1477-7819-11-70

**Published:** 2013-03-16

**Authors:** Peter A van Dam, Gerda Verheyden, Alessa Sugihara, Xuan B Trinh, Herman Van Der Mussele, Hilde Wuyts, Luc Verkinderen, Jan Hauspy, Peter Vermeulen, Luc Dirix

**Affiliations:** 1 Breast Unit, Department of Gynecology, Sint-Augustinus Hospital, Oosterveldlaan 24, Wilrijk, Belgium; 2 Medical student, University of Antwerp, Wilrijkstraat 10, Edegem, 2520, Belgium

**Keywords:** Breast cancer, Chemotherapy, Clinical pathway, Quality control, Radiotherapy, Surgery

## Abstract

**Background:**

Due to increasing the complexity of breast cancer treatment it is of paramount importance to develop structured care in order to avoid a chaotic and non-consistent management of patients. Clinical pathways, a result of the adaptation of the documents used in industrial quality management namely the Standard Operating Procedures, can be used to improve efficiency and quality of care. They also aim to re-centre the focus on the patient’s overall journey, rather than the contribution of each specialty or caring function independently.

**Methods:**

The effect of the implementation and prospective systematic evaluation of a clinical care pathway for the management of patients with early breast cancer in a single breast unit is evaluated over a long time interval (between 2002 and 2010). Annual analysis of predefined clinical outcome measures, service indicators, team indicators, process indicators and financial indicators was performed. Pathway quality control meetings were organized at least once a year. Systematic feedback was given to the team members, and if necessary the pathway was adapted according to evidence based literature data and in house pathway related data in order to improve quality.

**Results:**

The annual number of patients included in the pathway (289 *vs.* 390, *P* <0.01), proportion of patients with Tis-T1 tumors (42% *vs.* 58%, *P* <0.01), negative lymph nodes (44% *vs.* 58%, *P* <0.01) and no metastases at diagnosis (91.5% *vs.* 95.9%) has risen significantly between 2002 and 2010. Evolution of mandatory quality indicators defined by EUSOMA shows a significant improvement of quality of cancer care. Particularly, the proportion of patients having anti-hormonal therapy (84.8% *vs.* 97.4%, *P* = 0.002) and adjuvant chemotherapy according to the guidelines (72% *vs.* 95.6%, *P* = 0.028) increased dramatically. Patient satisfaction improved significantly (*P* <0.05). Progression free 4-year survival was significantly higher for all patients, for T1 tumors only and for T2-T4 tumors only, treated between 2006 to 2008 compared to between 1999 to 2002 and 2003 to 2005 (*P* = 0.006, *P* = 0.05, *P* = 0.06, respectively). Overall 4-year survival of the entire population treated between 2006 and 2008 was significantly better (*P* = 0.05).

**Conclusions:**

Although the patient characteristics changed over the years due to better screening, this clinical pathway and regular audit of quality indicators for the treatment of patients with operable breast cancer proved to be important tools to improve the quality of care, patient satisfaction and outcome.

## Background

Several publications have suggested that the quality of health care received by patients in the Western world does not always match the ideal care. In a survey of 30 health conditions ranging from osteoarthritis to breast cancer, McGlynn *et al.* observed that on average Americans received about half (54.9%) of the recommended medical care processes [1]. It has also been reported that in Europe there are wide differences in treatment offered to patients with breast cancer in terms of mastectomy and radiotherapy rates and the use of adjuvant chemotherapy and hormone therapy, which results in considerable survival differences [2,3]. These observations highlight a gap between optimal and actual care, that is, between what evidence has identified as recommended care and what patients actually receive [4]. They show that there is a worldwide need for tools to improve adherence to guidelines in the daily clinical practice.

High-quality services are essential to optimize treatment results of women with breast cancer. They can be achieved by accurate training, specialization, volume levels and a multidisciplinary approach, involving many different subspecialists, nursing staff and supporting staff members [5]. Due to the increasing complexity of breast cancer treatment and the teams involved, it is of paramount importance to develop structured care in order to avoid a chaotic and non-consistent management of patients.

A clinical care pathway is a methodology for the mutual decision making and organization of care for a well-defined group of patients during a well-defined period of time [6]. The aim of a clinical care pathway is to improve quality of care, reduce risks of unintended effects and death, increase patient satisfaction and improve efficiency of resource usage [7]. Clinical care pathways are developed by multi-professional teams, composed by different types of physicians, nurses, social workers and administrators, who manage disease processes and are responsible for patient care [8]. Continuous evaluation and follow-up of quality indicators should guarantee the effectiveness of a clinical care pathway. Five domains for evaluation can be distinguished: clinical outcome, service indicators, team indicators, process indicators and financial indicators [9]. A review on quality of cancer care, guidelines and clinical pathways demonstrated improvements in compliance to guidelines and evidence based medicine, and reduction of length of hospital stay, complication rates and financial costs [10]. The present paper describes the effects of the development, implementation and prospective systematic evaluation and adaptation of a clinical care pathway for the management of patients with operable breast cancer in a single breast unit over a long time interval (between 2002 and 2010).

## Methods

### The hospital setting

The Sint-Augustinus Hospital is a non-academic teaching hospital in Belgium. The Oncology Unit of the hospital is integrated in a local cancer network (Iridium Network), collaborates closely with Leuven University, Belgium, and is the second largest of Flanders. Every year up to 400 new breast cancer patients receive surgical treatment in this unit. Most patients are referred by local GP’s or the regional screening units.

Given that the multidisciplinary oncology team consisted of more than 40 physicians of different specialties, 50 members of the nursing staff, and 10 social workers and psychologists, a clinical pathway for the diagnosis, surgical treatment and decision making of adjuvant treatment in patients with operable breast cancer was developed in 2002 in an attempt to provide more uniform cancer care. Patients with a suspicious breast lesion obtain an appointment in the oncology outpatient clinic within two days, and by triple assessment a rapid diagnosis is achieved within five days. The postoperative multidisciplinary breast team meeting (held twice a week) outlines an individualized postoperative treatment plan based on the local cancer network guidelines (adaptation of the Sankt Gallen guidelines) [11-15]. Further treatment is carried out according to this plan if the patient agrees. Multidisciplinary follow-up is organized every 3 months, the first 3 years and biannually on the fourth and fifth year.

In 2005 a breast nurse was introduced to facilitate the patient’s journey through the multidisciplinary track and to be a gate-keeper of the clinical pathway. From January 2006 onwards immunohistochemical assessment of estrogen receptor, progesterone receptor and c-erbB-2 was standardized using FDA approved FARM DX and Herceptest Dako immunohistochemistry (Dako, Carpinteria, CA, USA) [16,17]. In January 2007 the Breast Clinic of the Sint-Augustinus Hospital was formally opened. It was organized according to European Society of Breast Cancer Specialists (EUSOMA) guidelines [18]. A breast multidisciplinary core team consisting of full-time breast surgeons, medical oncologists, radiotherapists, breast radiologists, histopathologists, breast care nurses, database manager, clinical geneticist, psychologists and reconstructive surgeons, was established. All patients with a new breast cancer diagnosis have a preoperative visit with a member of the core team. In addition a preoperative multidisciplinary meeting was introduced. Adaptations to the clinical pathway were made in 2008 in order to make it compatible with Flemish requirements for breast clinics. In 2008 the breast clinic of the Sint-Augustinus Hospital obtained its formal accreditation by the Flemish Government and EUSOMA [19]. Two new breast nurses were incorporated in the team in 2009, as our original breast nurse moved up in the administrative staff of the hospital. In 2010 our unit was the first unit in Belgium to receive a European Cancer Care Certificate, a quality label for breast cancer care. The pathology laboratory received an ISO2000 accreditation in 2010.

### Prospective data collection

As the hospital management wishes to guarantee high quality care by improving processes, since 2002, performance measurements have been documented systematically by care providers using an order communication, planning and result reporting system [20]. The nursing process is integrated in the clinical pathway “operable breast cancer”. The order communication system “patient care system” (PCS) is an IBM (New York, USA) mainframe based application working under Customer Information Control System (CICS) and using a Data Language One (DL/I) database. It has been completely adapted to the hospital requirements, by its own IT staff. It is developed as a level three electronic patient record as described by Brennan [21]. It contains clinical ward based applications including order communications and results reporting, multidisciplinary clinical pathways, electronic prescribing and drug administration.

The documentation process was improved by setting up a structured organization of data collection into the hospital informatics system, based on clear procedures discussed with care providers to make sure the data collection was achievable. As input of the many process parameters was very time consuming, it was decided to collect data and monitor them for the first half of every calendar year, so that in the second half of the year other care pathways could be assessed. Patient satisfaction was measured prospectively in 60 consecutive patients prior to discharge at the beginning of every year. A previously validated questionnaire developed by the Belgian-Dutch Clinical Pathway Network (BDCPN) on 19 different aspects of organization of care was used. It is based on a larger questionnaire developed by Chou *et al.*[22], and adapted and translated from English to Dutch. Questions were scored from 1 to 4 (1: dissatisfied; 2: more or less dissatisfied; 3: more or less satisfied; 4: satisfied). The responsibility for gathering correct data was identified and a structured follow-up was organized. Feedback and discussion of data and information of the responsible team was organized annually.

### Clinical pathway

The clinical pathway “operable breast cancer” was developed by the methodology described by the BDCPN [23]. The multidisciplinary team formulated the outcome parameters, critical indicators and key activities to deal with the patient’s condition. The clinical pathway is described by a time task matrix and integrated into the IT system PCS. The latest version of our clinical pathway operable breast cancer is attached to this manuscript (Additional file 1). Annual analysis of predefined clinical outcome measures, service indicators, team indicators, process indicators and financial indicators was performed. Pathway quality control meetings were organized at least once a year with the members of the core team, other medical providers and the hospital administrators. Systematic feedback was given to the team members, and if necessary the pathway was adapted according to evidence based literature data and in-house pathway related data in order to improve quality. Survival data were collected systematically for patients included in the in-house electronic patient records. If the patients were not followed in our hospital, their general practitioners were annually contacted to provide survival related information.

### Statistical analysis

The SPSS6 package was used for the statistical analysis of the data.

## Results

Characteristics of the patients treated between 2002 and 2010 are given in Table 1. As can be seen the number of patients included in the pathway and proportion of patients with small tumors, negative lymph nodes and no metastases at diagnosis has risen significantly over the years. Histological subtypes have remained the same, but the proportion of patients with hormone receptor negative tumors dropped significantly after the introduction of PharmDX immunohistochemical determination of hormone receptors in 2006.

**Table 1:** Patient characteristics between 2002 and 2010 in the clinical pathway “operable breast cancer”

**Indicator**	**2002**	**2003**	**2004**	**2005**	**2006**	**2007**	**2008**	**2009**	**2010**
Number of patients in pathway	140	130	130	108	164	176	146	169	183*
Percentage pTis-pT1 (%)	42	39	60	55	55	58	60	55	58*
Percentage pN0 (%)	44	52	46	48	51	58	57	52	58*
Percentage M1 at diagnosis	8.5	8.4	5.7	10	4.5	7.7	5.5	2.6	4.1*
Percentage IDC (%)	78	76	84	84	77	79	81	79	81*
Percentage ER negative (%)	26	21	34	31	19	20	23	18	18*

*: *P* <0.05 comparing 2002 *vs.* 2010.

IDC: invasive ductal carcinoma, ER: estrogen receptor.

Data were collected in the first semester of each year.

Table 2 shows the major clinical indicators between 2002 and 2010, indicating that the average length of hospital stay nearly halved and the proportion of breast conserving surgery, preoperative guide-wire localization for impalpable lesions and use of sentinel node biopsy increased significantly. Median duration of hospital stay was reduced for patients treated by mastectomy or breast conserving surgery from 9 and 4 days in 2002 to 7 and 2.5 days in 2010, respectively (*P* <0.01). The percentage of second surgery (to achieve free margins by additional local resection or mastectomy, or to perform a complete axillary dissection after a sentinel node biopsy which was negative during preoperative assessment but proved to contain metastatic cells at final pathological analysis) dropped from 25 to 10% (*P* <0.01). Sentinel node biopsy was introduced between 2005 and 2006, and its use remained stable over the years. Staging examinations have increasingly been performed preoperatively over the years. In 2009, when we had to incorporate two new breast nurses, a temporary drop of incompletely met discharge criteria was noted, which was corrected in 2010 after these nurses were further trained. “Completeness of discharge criteria” and “normal wound at discharge” were significantly better in the second half (2007 to 2010) of the evaluation period compared to the first half (2003 to 2005).

**Table 2:** Process indicators in the period 2002 to 2010 in the clinical pathway “operable breast cancer”

**Indicator**	**2002**	**2003**	**2004**	**2005**	**2006**	**2007**	**2008**	**2009**	**2010**
Number of patients in pathway	140	130	130	108	164	176	146	169	183 *
Total days of hospital stay (d)	991	913	980	651	936	872	730	848	745
Average hospital stay/patient (d)	7.0	7.0	7.5	6.0	5.7	4.9	5.0	5.0	4.1*
Breast conserving surgery (N, %)	60(43)	58(45)	58(45)	56(52)	86(53)	104(59)	82(56)	96(57)	106(58)*
Preoperative guide wire (N, %)	20(14)	26(20)	27(21)	23(21)	36(22)	28(16)	28(20)	35(21)	50(27)*
Sentinel node biopsy (N, %)	0(0)	7(5)	29(22)	48(44)	74(45)	82(46)	58(39)	64(38)	90(49)*
Preop staging tests (N, %)	74(53)	61(47)	56(43)	16(15)	38(23)	46(26)	19(13)	70(30)	22(12)*
All discharge criteria not Satisfactory (N, %)	NR	16(12)	19(15)	16(15)	18(11)	14(8)	7(5)	30(18)	16(9)
Wound not satisfactory at Planned discharge (N, %)	NR	4(3)	12(9)	10(9)	3(2)	3(2)	0(0)	5(3)	6(3)

N: number of patients, d: days; %: percentage; NR: not recorded.

*: *P* <0.05 comparing 2002 *vs.* 2010.

Data were collected in the first semester of each year.

**Figure 1:**
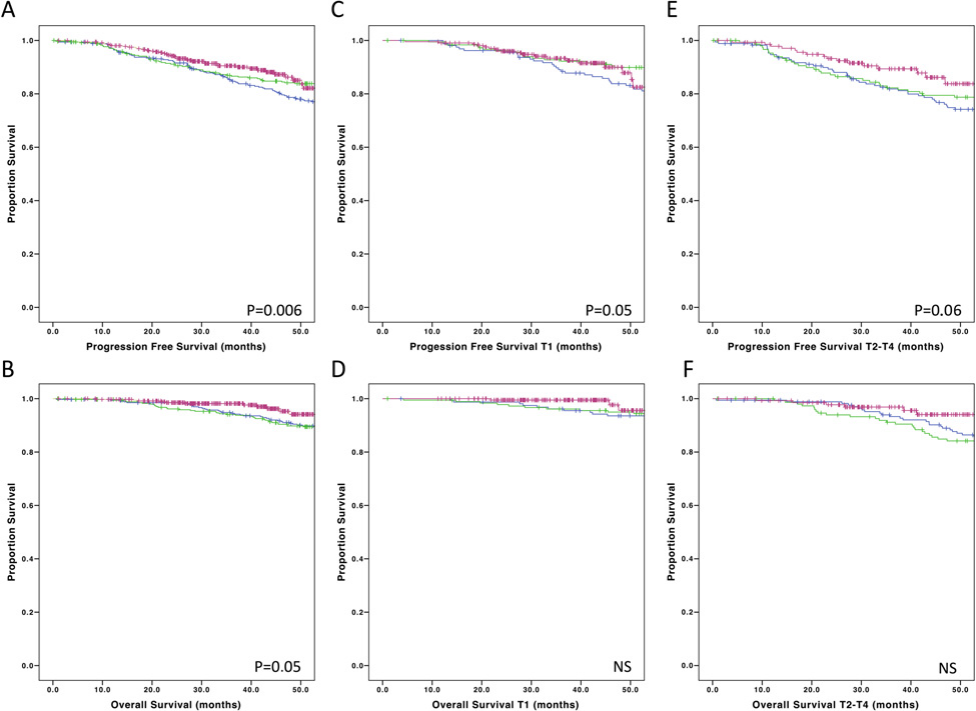
**Progression free and overall survival respectively of the entire population: (A) and (B); patients with T1 tumors only: (C) and (D); and patients with T2-T4 tumors only: (E) and (F).** Blue lines: patients treated in 1999-2002, green line: patients treated in 2003-2005, purple line: patients treated in 2006-2008.

Table 3 shows clinical indicators on postoperative day 1 which remain stable over the years. Of the EUSOMA indicators, ten are currently being monitored for the purpose of Cancer Care Certification by the use of the EUSOMA multi-institutional European Database. The time trend in results of these ten indicators in our unit is shown in Table 4. It can be seen that most of these indicators were significantly better in 2010 compared to 2003. EUSOMA criteria were met for all indicators in 2010. In Table 5 patient satisfaction is outlined. This clearly shows that patient satisfaction improved progressively over the years and was maximal in early 2009. There was a significantly higher patient satisfaction for 13 out of 19 parameters (*P* <0.05) measured in 2009 compared to 2003. The general level of patient satisfaction was very high. A slight drop in patient satisfaction was noted when the breast nurses changed at the end of 2009. Special teaching sessions were organized to improve this, and were clearly effective (Table 5). Progression free 4-year survival was significantly higher for all patients, for T1 tumors only and for T2-T4 tumors only, treated between 2006 and 2008 compared to between 1999 and 2002 and 2003 and 2005 (*P* = 0.006, *P* = 0.05, *P* = 0.06, respectively) (Figure 1). Overall 4-year survival of the entire population treated between 2006 and 2008 was significantly better (*P* = 0.05).

**Table 3:** Clinical indicators on the first postoperative day in the period 2004 to 2010 in the clinical pathway “operable breast cancer”

**Indicator**	**2004**	**2007**	**2008**	**2009**	**2010**	***P *value (2004–2010)**
Drainage first 24 hours (mL)	28.6	25.0	26.4	27.9	25.1	NS
VAS score >3 (N)	38(29)	19(10)	19	65	49	NS
Postoperative vomiting first 24 hours (N)	2(2)	2	3	9	11	NS
Postoperative fever (37.9°C) (N)	0(0)	1	3	0	0	NS
Norton score (average day 1)	20.0	19.9	19.9	19.8	19.9	NS

NS: not significant, N: number of patients.

Data were collected in the first semester of each year.

**Table 4:** Evolution of quality indicators as formulated by EUSOMA prospectively evaluated between 2003 and 2010 (data for first semester)

**Outcome measure**			**2003**		**2010**		***P *value**
	**EUSOMA**						
	**Minimum standard**	**Target**	**N**	**%**	**N**	**%**	
Positive preoperative cyto/histological diagnosis			77/130	59.7%	161/183	88.4%	0.0001
	**80%**	**90%**					
Operated invasive carcinoma for which hist. type, grading ER/PR, stage and size were recorded			106/112	94.6%	178/181	98.3%	0.1467
	**90%**	**95%**					
Operated non invasive carcinoma for which size, histological type and grading are recorded			5/7	71%	28/29	96.5%	0.4966
	**95%**	**98%**					
More than 9 lymph nodes removed when ALD performed (excluding sampling)			97/112	85.6%	67/70	95.7%	0.0434
	**95%**	**98%**					
Postoperative radiotherapy in M0 invasive CA with BCT			50/51	98%	105/108	97.2%	0.7588
	**90%**	**95%**					
BCT in invasive carcinoma with total size up to 30 mm (including DCIS component)			49/79	62%	86/104	82.6%	0.0016
	**70%**	**80%**					
BCT in carcinoma in situ up to 20 mm			3/7	43.8%	11/14	78.6	0.0016
	**70%**	**80%**					
Ductal carcinoma in situ without axillary dissection			6/7	85.7%	22/23	95.6%	0.3560
	**80%**	**90%**					
Hormonotherapy in endocrine sensitive invasive carcinoma			89/105	84.8%	151/155	97.4%	0.0002
	**80%**	**90%**					
Adjuvant chemotherapy in ER- (PT1c+ or N+) invasive Carcinoma			18/25	72%	22/23	95.6%	0.0280
	**80%**	**90%**					

N: number of patients; %: percentage of successful outcome measure/total number of cases; ER: estrogen receptor; PR: progesterone receptor; ALD: axillary lymph node dissection; BCT: breast conserving therapy; DCIS: ductal carcinoma *in situ*; significance level calculated with two-tailed *χ*^2^ test without Yates correction.

**Table 5:** Patient satisfaction in the clinical pathway “operable breast cancer” (systematically scored prospectively on 60 consecutive patients in the beginning of the year between 2003 and 2010)

**Satisfaction indicator**	**Year**								
**How satisfied were you about:**						**Significance level (*P *value)**			
	**2003**	**2004**	**2005**	**2006**	**2008**	**2009**	**2010**	**2003-2009**	**2009-2010**
Information on how to prepare your stay in the hospital									
	79	72	77	98	98	100	85	0.001	0.01
Information on the course of your stay (moment of admission until discharge)									
	85	85	87	93	97	98	92	0.05	NS
Information on how to prepare for a test or a treatment									
	85	87	90	98	98	100	96	0.01	NS
Explanation of the provided care before and during it was carried out									
	91	92	90	95	98	100	93	NS	NS
Information on your disease									
	87	90	98	98	97	98	98	NS	NS
Information which you received concerning the possible assistance after your discharge									
	77	90	97	95	98	95	98	0.007	NS
Uniformity of information you received from care providers									
	75	87	93	95	90	100	97	>0.001	NS
Smooth completion of your admission to the hospital									
	92	95	96	98	98	95	95	NS	NS
Consecution of investigative tests, interventions and general organization of care									
	92	92	93	98	100	100	92	0.05	NS
Waiting times during your hospital stay									
	75	100	97	85	90	97	82	0.002	NS
Complying of doctors and nurses with appointment during your stay									
	90	90	93	93	95	100	88	0.03	0.03
Hospital staff caring about a person, in a sense that you were not just a part of their job									
	70	95	97	97	100	100	98	>0.001	NS
Preparation you received to care for yourself after the moment of discharge									
	80	90	97	100	92	100	97	>0.001	NS
Degree in which you felt ready to leave the hospital at the moment of discharge									
	90	95	97	97	100	100	97	0.03	NS
Kindness of the care providers									
	90	97	100	100	100	98	98	NS	NS
Similarity of implementation of returning care									
	88	90	95	97	98	98	100	NS	NS
Teamwork among doctors, nurses, physiotherapists and other hospital staff									
	95	97	98	100	98	100	100	NS	NS
Guarantees of your privacy and dignity during your stay									
	90	92	97	97	100	100	100	0.03	NS
Initiative to keep your family well informed on your conditions									
	66	92	92	98	100	100	100	>0.001	NS

Significance level calculated by *χ*^2^ test; Figures are given as percentages of patients satisfied or very satisfied.

## Discussion

The clinical pathway concept appeared for the first time at the New England Medical Center (Boston, USA) in 1985 inspired by Zander and Bower [7]. Clinical pathways were a result of the adaptation of the documents used in industrial quality management, the Standard Operating Procedures, whose goals are to improve efficiency in the use of resources and to finish work in a set time. They also aim to re-centre the focus on the patient’s overall journey, rather than the contribution of each specialty or caring function independently. The difference between a pathway and a guideline is that a guideline defines the numerous acceptable treatment options that fall within the standard of care, whereas a pathway drives physicians toward a single treatment with predictable toxicities and minimal cost. Although the majority of patients are treated according to the pathway, it is possible for the team not to comply with the pathway for a particular case, but the reasons to do this have to be clearly documented.

Literature data on the use of clinical pathways for breast cancer care are limited to a few small studies. Kasahara and Tawaraya, in Japan, used five clinical pathways for the treatment of breast cancer patients [24]. They concluded that the clinical pathway brought standardization in their institution. The clinical pathway proved to be useful in coping with alternative operating methods, increased the use of adjuvant chemotherapy and increased the number of patients treated as outpatients. Santoso *et al.* showed, in a prospective analysis of a mastectomy clinical pathway over a seven month period in Singapore, that implementation of the clinical pathway improved consistency in patient treatment, the quality of patient outcome, and reduced costs of care and length of stay [25]. Hwang *et al.* found, in a retrospective analysis of 29 patients undergoing a transverse rectus abdominis breast reconstruction included in a clinical pathway compared to 40 similar non-pathway patients, that implementation of the reconstruction clinical pathway resulted in significant declines in length of stay and hospital costs but had no effect on complication rates [26]. Lee *et al.* showed that a clinical pathway for deep inferior epigastric perforans flap breast reconstruction reduced operating time and costs, and improved quality measures and staff satisfaction [27]. Ryhanen *et al.* demonstrated that clinical pathways can be used to increase patients’ knowledge of their disease and empowerment [28].

Goebel *et al.* concluded that clinical pathways can prevent malpractice lawsuits in breast cancer and radiation therapy [29]. In their retrospective analysis from the LexisNexis and Westlaw legal databases, they found that if physicians had adhered to clinical pathways, 49 out of 72 law-suits decided in favor of the plaintiff patient could have been avoided. In a recent project in Michigan it was suggested that the use of a breast cancer pathway reduces errors and costs, and increases efficiency [30]. These authors also found that patient satisfaction had increased since pathways were implemented. Particularly in an era of personalized medicine, clinical pathways are a tool to establish a model of care that drives oncologists towards evidence based medicine with measurable outcomes in order to achieve high quality patient outcomes at an affordable cost.

The present paper is the first to describe a prospective long-term analysis of the use of a clinical pathway to optimize management of large cohorts of patients with operable breast cancer. It clearly shows that a pathway can be a useful tool to assure uniform care and to improve adherence to guidelines. Continuous registration of quality indicators, treatment related data and regular feedback of the outcomes to the breast team improved quality of care significantly. Evaluation of the pathway data allows for corrective measures to improve care. When, for example, we noted more postoperative pain on day two in 2008 or more vomiting in 2010, a meeting was organized with the anesthetists, in order to improve the pathway. These corrective measures were effective and reduced these symptoms adequately. It should be mentioned that the exact “cocktail” of medication used for general anesthesia is not part of the pathway and depends on the preference of the anesthetist. A striking reduction in patient satisfaction was noted in 2009 when our original breast nurse moved up in the organization and was replaced by two full time breast nurses due to the increased workload. Particularly information for patients on how to prepare for their stay in the hospital, the waiting times during the hospital stay and compliance of doctors and nurses with appointments during the patients hospital stay was significantly lower. Special sessions were held with the breast nurses individually and with the entire core team, to bring this back to normal.

A recent Taiwanese study shows that when breast cancer patients are diagnosed and treated in complete accordance with widely accepted standards of care, they survive longer and have better outcomes [31]. This prospective study followed 1,378 newly diagnosed breast cancer patients from 1995 to 2001 in a single cancer hospital, tracking 10 indicators of care quality and assessing the progression of disease up to June 2007. Adherence to all 10 quality indicators by patients was associated with better overall (HR: 0.46; 95% CI: 0.33 to 0.63) and progression-free survival (HR: 0.51; 95% CI: 0.39 to 0.67). Adherence to either the four treatment indicators, or the six diagnostic indicators by patients was also associated with a significant improvement of survival. In the present study 4-year progression free survival was significantly better in the cohort of patients treated in 2006 to 2008 compared to 2004 to 2005 and 2002 to 2003. Similar results were obtained after data were stratified for T1 tumors only and T2-T4 tumors. Although our survival results can partly be explained by an evolution in the case mix, with considerably more patients with small tumors and negative lymph nodes in more recent years, better adherence to guidelines is likely to be beneficial for the outcome of the patients. In 2010 more than 97%, 97% and 95% of patients had state of the art adjuvant radiotherapeutic, anti-hormonal or cytostatic treatment, respectively, when indicated (according to the Sankt Gallen guidelines), compared to 98%, 85% and 72% in 2003. A benchmarking system of the quality of breast cancer care by a nationwide voluntary collaborative network of breast centers in Germany showed similar results [32]. Monitoring pre-defined quality indicators significantly improved preoperative histological confirmation of diagnosis (58% in 2003) to 88% in 2008, appropriate endocrine therapy in hormone receptor positive patients (27 to 93%), appropriate radiotherapy after breast conserving therapy (20 to 79%) and appropriate radiotherapy after mastectomy (8 to 65%).

## Conclusions

EUSOMA has started a voluntary certification process to assess the clinical performance in dedicated European units [5,18,33]. So far, 32 breast units in Europe have been recognized to comply with the requirements requested by EUSOMA and other European Union guidelines on the basis of information collected by a questionnaire and by a site visit carried out by an independent team of breast cancer experts. A set of quality indicators was defined by experts from different disciplines based on a literature review. These clearly defined quality parameters, continuous internal audit and external social control by means of a site visit are of paramount importance to optimize adherence to evidence based guidelines and treatment results. The present data shows that data collection results in knowledge which can be used for the benefit of the patients. Clinical pathways for breast cancer have proven to be useful tools to implement better breast cancer care.

## Abbreviations

BDCPN: Belgian-Dutch Clinical Pathway Network; EUSOMA: European Society of Breast Cancer Specialists; PCS: Patient Care System

## Competing interests

None of the authors has any financial of personal relationships with other people or organizations that could inappropriately influences this work.

## Authors’ contributions

PD wrote the article and played a major role in developing and auditing the pathway; GV and HVM played a major role in developing the pathway and analyzing the data; AS translated the clinical pathway operable breast cancer from Dutch to English; BT performed the survival analysis; HW was responsible for part of the data management; LV, JH, PV and LD played a role in developing and auditing the pathway and edited the manuscript. All authors read and approved the final manuscript.

## Supplementary Material

Additional file 1 Operable breast cancer. Click here for file
