# Supplementary material for: A dynamic clinical pathway for the treatment of patients with early breast cancer is a tool for better cancer care: implementation and prospective analysis between 2002–2010
Source: World J Surg Oncol. 2013 Mar 16;11:70. doi: 10.1186/1477-7819-11-70 (PMC3623911; doi:10.1186/1477-7819-11-70)
Supplement: Additional file 1 — Operable breast cancer. [file 1477-7819-11-70-S1.docx]

| **Operable breast cancer** | | | 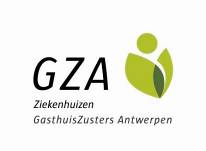  Operaties  Zorg |
| --- | --- | --- | --- |
| **Clinical pathway only valid in :**  Campus : **SA**  Specialty : **Gynaecology**  **Gynaecologie** | Date of implementation: 18/06/2012  Date of expiration : 18/07/2013  Document Manager: Verheyden Gerda  Attending Physician : van Dam Peter | |  |
|  |  |  | Versie: 2.0 |
| **Objective:**   - Pre-operative: patient is in optimal physical and psychosocial condition before the intervention- optimal planning of clinical care - Postoperative: patient receives high quality and safe post-operative care and aftercare | | | |
| **Inclusion criteria**  *Comment: the hospital ward is notified by means of registration forms about the patient’s admission to the clinical pathway*  Patiënts with primary operable breast cancer | | **Exclusion criteria**  Metastases | |
| **Pre-operative discharge criteria**   - Physician - Clinical staging / clinical TNM - Intervention date within 15 days of diagnosis - Trusting relationship - Explanation future prospects - Consult oncologist - Breast care nurse: - Consult at time of diagnosis/ pre-operative - Check understanding of information - Social services - Pre-operative consult for high risk patients - Psychologist - Pre-operative consult for risk patients | | **Assurance: Evaluation criteria**   - Clinical indicators - Tripple assessment - Eusoma indicators: 10 compulsory indicators - Process indicators - Stagings test before intervention - True cut biopsy within 48h of 1st consult - Pre-operative Multidisciplinary Oncological Committee (MOC) - Diagnosis within 5 labor days - Pre-operative consult breast care nurse - Financial indicators - Number of patients - Average length of hospital stay - Patient satisfaction | |
| **Link to:**   - Clinical pathway phase - [link](file:///K:/JCI%20zorgpaden/7%20fasen%20model.docx) - Definitions - [link](file:///K:/JCI%20zorgpaden/Definities.docx) | |  | |

| **Admission phase** | **Day -14 until day -10** | **Day -10 until day -7** | **Day -6 until day -2 / 1** |
| --- | --- | --- | --- |
|  |  |  |  |
| **Critical indicators** | Consult within 24 h | True cut within 48 h | Time 1^st^ consult - diagnosis |
| **Key interventions** | Consult breast clinic | True cut | Pre-operative MOC |
|  |  |  |  |
| **Medical Care** | Breast clinic consultation  Interpretation bilateral mammography – ultrasound   - Breast MR Imaging - Appointment oncological center - Scheduling biopsy | Communicate diagnosis  Communicate further planning  Pre-operative diagnostic examinations | Results staging tests  Scheduling intervention  Result pre-operative MOC |
| **Medication** | Pre-admission medication | Pre-admission medication | Stop oral anti-coagulation day -7 |
| **Nursing care** |  |  |  |
| **Nursing specialist** | - Introduction breast care nurse - Scheduling true cut biopsy - Scheduling tests - Emotional support | Present at time of diagnosis communication   - Review diagnosis/ prognosis - Team introduction   Illustrate information leaflet  Emotional support  Appointments stagings tests   - Liver ultrasound - Chest Xray - Bone scan - Venipuncture CA 15.3 | Scheduling   - Sentinel - Guide wire biopsy   Referral self-help group  Emotional support  Discussion/review surgical intervention  Treatment  Clincal pathway |
| **Oncocoach** |  | Analysis health performance score using the “health meter”  Liaison function |  |
| **Social services** |  | Optional consult |  |
| **Psychology** |  | Available for consult |  |
| **Nutrition** | Normal diet or pre-admission diet | Normal diet or pre-admission diet | Normal diet or pre-admission diet |
| **Physiotherapy/Mobilisation** |  |  |  |
| **Tests** | Health performance score (Health meter) | True cut biospsy  Vacuum assisted biopsy in case of microcalcifications | Pre-operative tests  [link](http://portaal.gza.be/kennisbank/procedureboeken/KlinischeProcedures/Documents/Preoperatie%20onderzoek%20nieuwe%20richtlijnen%2020060424.pdf)  Staging tests Liver ultrasound   - Chest Xray - Bone scan - Venipuncture CA 15.3   Sentinel lymph node |
| **Material** |  | Information leaflet |  |
| **Information/Education** |  | Intervention  Aftercare  Wire-guided localization / sentinel  MR Imaging/ mammography  Breast cancer |  |
| **Discharge preparation** |  |  | Check discharge indicators  Pre-operative MOC concluded  Date of intervention known  Check pre-admission |

| **Inclusion criteria operative phase**  *Comment: the hospital ward is notified by means of registration forms about the patient’s admission to the clinical pathway*  Patiënts with primary operable breast cancer | **Exclusion criteria operatieve fase**  Metastases |
| --- | --- |
| **Pre-operative discharge criteria**   - Physician - Had an informative conversation with the patient, patient is informed about aftercare and time of discharge. - Has seen cytology results - Follow-up appointment - Appointment for suture removal - Oncologist (cooperating doctors) - 2nd contact moment about aftercare - Appointment adjuvant medical treatment planning visit - Breast care nurse: - Coordination and planning further course - Checklist   - patient understood aftercare information  - appointment adjuvant medical treatment planning visit  - information about social services/psychologist  - physiotherapy appointment  - self-care/ information about prosthesis  - information about self-help groups   - Information about individual therapy, counseling programs etc. - Check planning visit - Social services - Organized homecare services if necessary - Information - Psychologist - Pre-operative consult if risk patient | **Assurance: evaluation criteria**   - Clinical indicators - Tripple assessment - Eusoma indicators: 10 compulsory indicators - Process indicators - Stagings test before intervention - True cut biopsy within 48h of 1st consult - Pre-operative Multidisciplinary Oncological Committee (MOC) - Diagnosis within 5 labor days - Pre-operative consult breast care nurse - Financial indicators - Number of patients - Average length of hospital stay - Patient satisfaction |

| **Admission phase** | **Day -1** | **Day O** | **Day 1** | **Day 2** |
| --- | --- | --- | --- | --- |
|  |  |  |  |  |
| **Critical indicators** |  | Pain core < 3 | Patient mobilisation  Pain score < 3 | Pain score < 3 |
| **Key interventions** | Admission  Sentinel lymph node | Surgical intervention | Arm mobilisation  Patient mobilisation | Drain removal  Social services  Prosthesis information |
|  |  |  |  |  |
| **Medical care** | Pre-operative anesthesia consult | Surgical intervention |  | Check bandage |
| **Medication** | Pre-admisson medication | **Pre-admission medication** (optional)  -------------------------------------  **Pre-operative medication**  Temesta  --------------------------------------  **Fluid management**  Standing order remaining fluid  1l Hartmann IV over 12 h  **------------------------------------**  **Pain management**  Paracetamol 1g 100ml Perf. Fresenius 100ml/30min 1x/hu 100 ml IV  *Escape*  Tramadol100mg ampul 1x/perf 1 ampul IV + Litican 2ml ampul 1x/perf 1 ampul IV(L) + Natriumchloride 0,9% 100ml Minibag 100ml/30min 1x/6h IV  --------------------------------------  **PONB**  Protocol PONB  [link](http://portaal.gza.be/kennisbank/procedureboeken/VerpleegkundigProcedureboek/Algoritme%20voor%20PONB%20(postoperatief%20nausea%20en%20braken).docx)  -------------------------------------- | **Pre-admission medication** restart  --- --------------------------------  **Pain management**  Paracetamol 1g tablet  1x/6h standing order  --------------------------------------  **PONB**  Protocol PONB  [link](http://portaal.gza.be/kennisbank/procedureboeken/VerpleegkundigProcedureboek/Algoritme%20voor%20PONB%20(postoperatief%20nausea%20en%20braken).docx)  --------------------------------------  20h:  Sleep medication if needed  Loramet 2 mg PO < 60 y  Loramet 1 mg PO >60 y | **Pre-admission medication**  --------------------------------------  **Pain management**  Paracetamol 1g tablet  1x/6h on demand  --------------------------------------  20h:  Sleep medication if needed  Loramet 2 mg PO < 60 y  Loramet 1 mg PO >60 y |
| **Nursing care** | Check ambulant tests  - Mammography/ US  - Chest Xray  - Venipuncture  - ECG  --------------------------------------  Nurse patient history:  - Norton Score+ length + weight  - Patient profile  - Pre-admission medication  - Diet | **Pre-opertative preparation**  - Check ID bracelet  - Operation gown  - Identification tags  - Removal:  dental prostheses, piercings, jewelry    --------------------------------------  **Hygiene**  - Shower or washbasin  --------------------------------------  **Vital parameters**  - Blood pressure, pulse  --------------------------------------  **Post-operative checks**  Every 2h during 8h  Thereafter, every 4h during 8h  - IV drip  - VAS Nausea  - VAS Pain  - Consciousness  **Post-operative parameters**  Every 2h during 8h  Thereafter, every 4h during 8h  - Blood pressure, pulse  **Post-operative wound care**  Every 2h during 8h  Thereafter, every 4h during 8h  - Check drain: color, volume, air vacuity  **Secretions**  Check spontaneous miction | **Vital parameters**  Blood pressure and pulse before morning nursing- Tempature 2x/day and when necessary  --------------------------------------  **Hygiene**  - Bed bath  - Washing private parts  - Assistance with wash basin  --------------------------------------  **Check 2x/day and if needed**  - VAS pain  - VAS nausea  - Consciousness  --------------------------------------  **Wound care**  Check 2x/day and if necessary  - Bandage  - Drain  --------------------------------------  **Mobility**  First: standing up and walk in the room | **Vital parameters**  Blood pressure and pulse before morning nursing- Tempature 2x/day and when necessary  --------------------------------------  **Hygiene**  - Washing private parts  - Assistance with wash basin  --------------------------------------  **Check 2x/day en if needed**  - VAS pain  - VAS nausea  - Consciousness  --------------------------------------  **Wondzorg**  Controle 2X/d en z.n.:  - Sterile dry dressing  - Remove drain if < 20 ml /24 h  -------------------------------------- |
| **Nursing specialist** | Information:  - surgical intervention  - care pathway  Emotional support  Referral: social services, psychologist | Information: intervention date  Emotional support | Information:  - course of the surgery  - wound  Emotional support | Information:  - self-help groups  - prostheses  - revalidation |
| **Oncocoach** |  | Contact breast nurse |  |  |
| **Social services** |  |  | Optional after contacting breast nurse:  Contact social services:  - Delineate domestic setting  - Professional status  - Hospitalisation insurance |  |
| **Psychology** | Optional consult | Optional consult | Optional consult | Optional consult |
| **Nutrition** | Normal diet | Fasting  Fluids allowed 6h after surgery | Normal diet | Normal diet |
| **Physiotherapy/**  **Mobilisation** |  |  | Arm mobilisation after axillary lymph node dissection (ALND) |  |
| **Tests** |  |  |  |  |
| **Material** |  |  |  | Breast prosthesis  Information leaflet |
| **Information/Education** |  |  |  |  |
| **Discharge preparation** |  |  |  | Appointment for follow-up  Adjuvant treatment planning visit |

| **Admission phase** | **Day 3** | **Day 4** | **Day 5** | **Day 6** |
| --- | --- | --- | --- | --- |
|  |  |  |  |  |
| **Critical indicators** | Pain < 3 | Pain < 3 | Pain < 3  Anatomical pathology report |  |
| **Key interventions** |  | Psycho-social consultation:  - breast care nurse  - psychologist  - social services | MOC |  |
|  |  |  |  |  |
| **Medical care** | Informative conversation  Planning discharge date | Informative conversation  Planning discharge date | Informative conversation  Planning discharge date  Follow-up appointment |  |
| **Medication** | Pre-admission medication  --------------------------------------  **Pain management**  Dafalgan Forte 1g effervescent tablet 1x/6u  1 tablet on demand  --------------------------------------  20u:  Sleep medication if needed:  Loramet 2 mg PO < 60 y  Loramet 1 mg PO >60 y | Thuismedicatie  --------------------------------------  **Pain management**  Dafalgan Forte 1g effervescent tablet 1x/6u  1 tablet on demand  --------------------------------------  20u:  Sleep medication if needed:  Loramet 2 mg PO < 60 y  Loramet 1 mg PO >60 y | Thuismedicatie  --------------------------------------  **Pain management**  Dafalgan Forte 1g effervescent tablet 1x/6u  1 tablet on demand  --------------------------------------  20u:  Sleep medication if needed:  Loramet 2 mg PO < 60 y  Loramet 1 mg PO >60 y |  |
| **Nursing care** | **Vital parameters**  - Blood pressure and pulse before morning nursing  - Temperature 2x/day and if needed  --------------------------------------  **Hygiene**  - Assistance with washbasin  --------------------------------------  **Check 2X/d and if needed**  - VAS pain  - VAS nausea  - VAS fatigue  - Consciousness  **Wound care**  Check 2x/day and if needed:  - Sterile dry dressing  - Remove drain if < 20ml/24h | **Vital parameters**  - Blood pressure and pulse before morning nursing  - Temperature 2x/day and if needed  --------------------------------------  **Hygiene**  - Assistance with washbasin  --------------------------------------  **Check 2X/d and if needed**  - VAS pain  - VAS nausea  - VAS fatigue  - Consciousness  **Wound care**  Check 2x/day and if needed:  - Sterile dry dressing  - Remove drain if < 20ml/24h | **Vital parameters**  - Blood pressure and pulse before morning nursing  - Temperature 2x/day and if needed  --------------------------------------  **Hygiene**  - Assistance with washbasin  --------------------------------------  **Check 2X/d and if needed**  - VAS pain  - VAS nausea  - VAS fatigue  - Consciousness  **Wound care**  Check 2x/day and if needed:  - Sterile dry dressing  - Remove drain if < 20ml/24h |  |
| **Nursing specialist** | Information:  - surgical intervention  - hospitalisation course  Emotional support  Contact psychologist/ social services | Information  - surgical intervention  - additional tests  - Emotional support | Information:  - course of the surgery  - wound  - self-help groups (+perhaps addresses)  Emotional support  Adjust prothesis in case of mammectomy  - Follow-up consult |  |
| **Oncocoach** |  |  |  |  |
| **Social services** | Optional consult after visit of the breast nurse |  |  |  |
| **Psychology** |  | Psychosocial consultation 1X/week |  |  |
| **Nutrition** | Normal diet | Fasting  Fluids allowed 6h after surgery |  |  |
| **Physiotherapy/ Mobilisation** |  | Post-operative bedrest, depending on vital parameters  Systematic application for physiotherapy in case of ALND | Start arm mobilisation in bed in case of ALND  Continue arm mobilisation |  |
| **Tests** |  |  |  |  |
| **Material** |  |  | Naboram visit (patient supporting group involved in fitting breast prothesis)  Providing written information on chemotherapy / radiotherapy optional |  |
| **Information/Education** |  |  | Breast prosthesis  Wound care  Hygiene |  |
| **Discharge preparation** |  |  | Discharge criteria satisfied |  |

| **Multidisciplinary team** | | | | | | | |
| --- | --- | --- | --- | --- | --- | --- | --- |
|  | | | | | | | |
|  | **Departments** | **Name** |  |  |  | **Departments** | **Names** |
|  |  |  |  |  |  |  |  |
| **Physicians** | Medical coordinator  Gynaecologic oncology | P. van Dam |  |  |  |  |  |
|  | Gynaecologic oncology | L. Verkinderen |  |  |  |  |  |
|  | Gynaecologic oncology | J. Hauspy |  |  | **Nurse** | Gynaecology | C. Martens |
|  |  |  |  |  |  | Gynaecology | N. Becuwe |
|  | Anesthesiology | Van Houwe |  |  |  |  |  |
|  |  |  |  |  | **Psychosocial** | Psychology | S. Van Gaelen |
|  | Anatomic pathology | P. Vermeulen |  |  |  |  |  |
|  |  | C. Colpaert |  |  | Nurse | Social services | T. De Backer |
|  |  |  |  |  | Social worker | Social services | E. De Jongh |
|  | Radiologiy | A. De Schepper |  |  | Social worker | Social services | K. Moerman |
|  |  | A. Bernaerts |  |  | Nurse | Social services | I. Deschacht |
|  |  |  |  |  |  |  |  |
|  | Medical oncology | L. Dirix |  |  | Breast care nurse | Nursing staff | A. Van As |
|  |  | A. Prové |  |  | Breast care nurse | Nursing staff | C. Guiette |
|  |  |  |  |  | Breast care nurse | Nursing staff | C. Dhaenens |
|  | Radiotherapy | C. De Pooter |  |  |  |  |  |
|  |  | G. Buelens |  |  | **Paramedical** |  |  |
|  |  | R. Weytjens |  |  | Physiotherapist | Physiotherapy | R. Hofkens |
|  |  |  |  |  | Physiotherapist | Physiotherapy | K. Proost |
|  | Reconstructive surgery | J. Vanoorbeek |  |  |  |  |  |
|  |  |  |  |  | Oncology dietitian | Oncology nutrition | J. Liekens |
|  | Physiotherapy | K. Deckers |  |  |  |  |  |
|  |  |  |  |  | **Datamanager** | Cancer registration | I. Daniels |
|  | Genetics | M. De Smedt |  |  |  |  |  |
|  |  |  |  |  | **IT-coördinator** | IT | M. Mertens |
|  | Psychiatry | A. Dewit |  |  |  |  |  |
|  |  |  |  |  | **Clinical pathway coördinator** | Cell management support | G. Verheyden |
|  | Gynaecology | J. Van Wiemeersch |  |  |  |  |  |
|  |  |  |  |  | **Extern** | Naboram |  |

| **Approval** |
| --- |
| Approved by:  van Dam Peter  [Goedgekeurd Door 2]  [Goedgekeurd Door 3]  [Goedgekeurd Door 4]  [Goedgekeurd Door 5] |
| Ref. nr.: - |
